# Manipulation of Dietary Intake on Changes in Circulating Testosterone Concentrations

**DOI:** 10.3390/nu13103375

**Published:** 2021-09-25

**Authors:** Amit Zamir, Tavor Ben-Zeev, Jay R. Hoffman

**Affiliations:** Department of Physical Therapy, Faculty of Health Sciences, Ariel University, 40700 Ariel, Israel; amitza@ariel.ac.il (A.Z.); tavorbenzeev@gmail.com (T.B.-Z.)

**Keywords:** androgens, macronutrients, micronutrients, diet, aromatase activity

## Abstract

Elevations in the circulating concentration of androgens are thought to have a positive effect on the anabolic processes leading to improved athletic performance. Anabolic-androgenic steroids have often been used by competitive athletes to augment this effect. Although there has been concerted effort on examining how manipulating training variables (e.g., intensity and volume of training) can influence the androgen response to exercise, there has been much less effort directed at understanding how changes in both macronutrient and micronutrient intake can impact the androgen response. Thus, the focus of this review is to examine the effect that manipulating energy and nutrient intake has on circulating concentrations of testosterone and what the potential mechanism is governing these changes.

## 1. Introduction

Testosterone, together with its potent metabolite, dihydrotestosterone (DHT), are the principal androgens in the circulation of mature male mammals, including humans. They are important hormones for various biological processes and are vital for the development and maintenance of secondary male characteristics. They are also crucial for reproductive functions, body composition, and muscle and bone health [1,2].

As the primary anabolic steroid, testosterone promotes an increase in protein production as well as stimulating both anabolic and anti-catabolic functions in skeletal muscle and neuronal tissue leading to increased muscle strength, power, endurance, and hypertrophy in a dose-dependent manner [3]. Testosterone is also responsible for the mass, density, and strength of bone. As for its androgenic effects, testosterone mediates the development of male primary and secondary male characteristics such as sexual organ growth, deepening of the voice, and growth of facial and body hair [4].

Structurally, testosterone has a characteristic four ring C18 steroid structure and is synthesized from cholesterol through an enzymatic multistep process primarily within the Leydig cells (~95%), which are located in the interstitium of the testes. The adrenal glands also produce small amounts (~5%) of androgens [5]. In women, testosterone is produced in much smaller amounts, primarily from the adrenal glands and the ovaries [2,6]. There are two metabolic pathways, the progesterone (delta-4) and dehydroepiandrosterone (DHEA) (delta-5) pathways [6]. Once synthesized, testosterone is secreted into the bloodstream and delivered to target tissues [7]. In the blood, most testosterone is transported bound to several proteins, mainly serum albumin and sex hormone-binding globulin (SHBG). A small amount is transported unbound, referred to as free testosterone (FT) [8]. FT is the active form of testosterone while protein-bound testosterone is inactive [9]. When testosterone reaches its target tissues it diffuses through the cells’ fatty membrane, where it can interact with its receptor stimulating its biological effects or it can be reduced into DHT by the cytoplasmic enzyme 5α-reductase, which is highly expressed in male reproductive organs, skin, and the brain [8].

Testosterone is synthesized under control of the hypothalamic-anterior pituitary-gonadal axis. Gonadotropin-releasing hormone (GnRH), released from the hypothalamus, stimulates the release of luteinizing hormone (LH) from the anterior pituitary gland into the circulation. LH then stimulates the Leydig cells within the testes to synthesize testosterone. Increasing circulating concentrations of testosterone will result in an inhibition of the release of the gonadotrophins (GnRH and LH) via a negative feedback loop mechanism. GnRH functions under the control of several hypothalamic neuropeptides [5].

The classical biological effects of androgens are primarily mediated by FT binding to the androgen receptor (AR). DHT binds to the same AR even more strongly than testosterone, so that its androgenic potency is about five times more potent than testosterone [10]. The AR complex undergoes a structural change that allows it to move into the cell nucleus and bind directly to specific nucleotide sequences of DNA, resulting in the transcription of certain genes. The AR complex itself serves as a transcription factor [7,11]. Testosterone can also be converted to estradiol (E2) by the aromatase enzyme and then activate certain estrogen receptors. Bone, adipose tissue, and the brain are tissues in humans where the primary effect of testosterone is via aromatization to E2 [4,12]. The enzyme aromatase is a member of the cytochrome P450 enzyme superfamily that catalyzes the conversion of androstenedione and testosterone to the aromatic estrogenic steroids estrone and estradiol, respectively [9]. These are the last key steps in the catalyzation of androgens into estrogens, hence inhibition of aromatase activity can elevate androgen concentrations. There are numerous natural substances that have been scientifically tested or suggested to inhibit aromatase activity, alongside pharmaceutical compounds whose non-medical use is considered illegal [2,6].

Manipulation of testosterone concentrations without the use of anabolic steroids has been a highly investigated topic because of the known effect testosterone has on enhancing athletic performance [2,6]. While the use of androgens in competitive athletics is illegal, it has not stopped the search for “natural” ways to increase testosterone concentrations. In this review, we will examine the specific effect of various macronutrients and micronutrients on enhancing circulating testosterone concentrations at rest and during exercise. In addition, we will also discuss the role of low energy availability, a growing condition in the athletic population, and its effect on testosterone concentrations. While other reviews have previously discussed the effect of nutrition on testosterone status [13,14,15,16], most of these papers have examined the role of single nutrient (macro/micro) or food/food groups. To the best of our knowledge, this review is the first to focus on the nutrient influence on circulating testosterone concentrations. Its purpose is to provide an evidence-based assessment of how specific nutrients found in the diet or specific dietary manipulation can enhance the androgen response, with the assumption being that elevation in circulating androgens will enhance the anabolic response and potentially improve exercise performance.

### 1.1. Natural Product Extracts and Aromatase Inhibition

**Figure 1:**
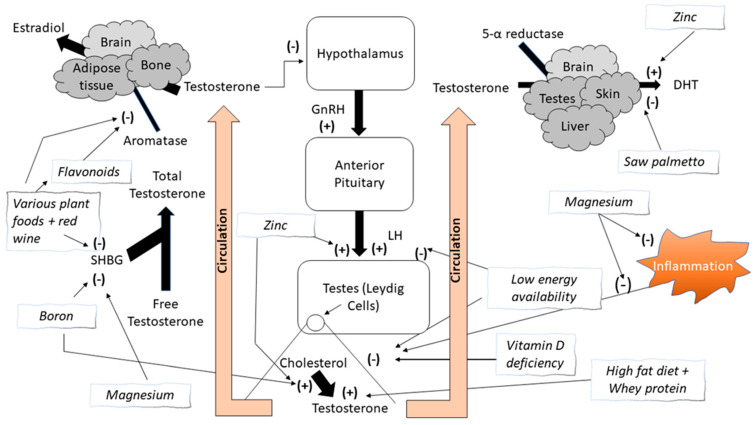
The effect of various food groups, macronutrients, and micronutrients on testosterone circulation and its proposed mechanisms. (+) = Increases or stimulates; (−) = Decreases or inhibits; DHT = Dihydrotestosterone; LH = Luteinizing hormone; SHBG = Sex hormone binding globulin.

Extracts of many natural sources and nutrients, part of them used by traditional medicine, have been tested and found to possess aromatase inhibition activity. Figure 1 provides an overview of the effect that these various compounds have on aromatase activity. These natural sources include *Brassaiopsis glomerulata* from the *Araliaceae* plant family (i.e., ginseng). This species has several reported medicinal uses in south and southeast Asia, such as treatment of rheumatism and back pain, aiding in digestion and alleviating constipation, treating bone fractures and sprains, and other health issues [17]. Balunas and colleagues [18] reported strong aromatase inhibition of the hexane extract of *Brassaiopsis glomerulata*, coupled with the possibility of a favorable safety profile. Cycads have been used by numerous cultures for medicinal purposes such as the treatment of certain tumors, treating wounds, hemorrhoids, and more [19]. Kowalska and colleagues [19] reported five different species of cycad folia to have aromatase inhibition activity. These investigators reported that several other plant species also possess aromatase inhibition capabilities similar to the potent aromatase inhibitor, l0-propargylestr.4-ene-3,17-dione, including members of the *Loranthaceae, Santalaceae* and *Zingiberaceae* families, all flowering plants [20].

*Isodon excisus* var. coreanus, a member of the *Lamiaceae* family, is one of the endemic plants in Korea, and has been used for the treatment of the anorexia, indigestion, stomachache, inflammation, and esophageal carcinoma [21]. Jeong and colleagues [22] reported four compounds isolated from this plant, including diethyl ether extract, inflexin, ursolic acid, and ursolic acid 3-O-acetate that have significant aromatase inhibitory activity. Lee and colleagues [23] reported that extracts from *Euonymus alatus*, a member of the *Celastraceae* family, also has potent aromatase inhibition.

The health benefits of red wine have been well-examined, and in recent decades studies have reported that red wine consumption has antioxidant, lipid regulating, and anti-inflammatory effects [24]. Interestingly, red wine has also been reported to result in aromatase inhibition. There have been five red wine varieties that have been reported to have aromatase inhibition activity, with the most active being cabernet sauvignon [25,26,27]. Consistent with the anti-aromatase benefits associated with red wine, Kijima and colleagues [28] reported that grape seed extract is a potent aromatase inhibitor and suppressor of aromatase expression. Shufelt and colleagues [29] reported that red wine consumption resulted in significantly higher FT concentrations and lower SHBG and E2 concentrations in women. This may have important implications regarding breast cancer risk. Interestingly, red wine was significantly more beneficial than white wine. Whether this same response is seen in men is not well understood.

Other food groups have also been reported to have anti-aromatase activity. White button mushrooms are known for their innate immune response enhancement [30,31] and their role in fat metabolism resulting in a decrease in cholesterol concentrations [31]. Grube and colleagues [32] reported that white button mushroom consumption can inhibit aromatase activity and breast cancer cell proliferation in women. However, there is limited evidence of this in men. Red clover flower extract, often used to improve hair and skin texture [33,34], has also been reported to inhibit 5-α-reductase activity [35]. Interestingly, Almstrup and colleagues [35] reported that red clover flowers inhibit aromatase activity at low concentrations, but become estrogenic at higher concentrations, resulting in a U-shaped dose–response curve.

Balunas and colleagues [36] reported that an extract from a tropical fruit called mangosteen had strong inhibitory capabilities of the aromatase enzyme. Although this research team was primarily focused on the role that this fruit had on preventing or treating breast cancer, others have examined the ergogenic effect of this fruit. Konda and colleagues [37] examined the ergogenic effects of *Garcina mangostana* on both rodents and humans. Mice supplemented with *Garcina mangostana* experienced significantly greater swim times, swim distance, and grip strength than control mice. In addition, resistance-trained men were provided 42 days of the supplement (800 mg·day^−1^) and experienced significant greater increases in maximal strength in the bench press and leg press exercise compared to a placebo-controlled group. In addition, the group consuming the supplement performed significantly more repetitions on the leg extension exercise and realized significantly greater increases in lean body mass and arm circumference than the placebo group. Significant improvements were reported in FT concentrations in the supplement group compared to the placebo group. To the best of our knowledge, this is the only study that has examined the anabolic effects of *Garcina mangostana* in humans. Further research on this extract appears warranted.

Sultan and colleagues [38] reported that an extract from saw palmetto (*Serenoa repens*), a type of a palm tree, decreases in vitro 5α-reduction of androgens. Saw palmetto has been used to treat urinary tract infections and prostatic diseases [39]. Sudeep and colleagues [39] investigated 12 weeks of saw palmetto oil supplementation on androgen-deficient men and reported significant increases in both FT concentrations and quality of life in the supplemented men compared to placebo-treated men. Others have reported that two weeks of saw palmetto supplementation can significantly increase total testosterone and decrease DHT and E2 concentrations using both low (800 mg·day^−1^) and high (2000 mg·day^−1^) doses [40]. Although the efficacy of saw palmetto has been demonstrated in androgen-deficient men, there is limited data on younger, eugonadal men.

There are numerous other plant extracts from collards, tomato leaves, tea, coffee, cocoa, kale, potato leaves that have been reported to have high aromatase inhibition activity [41]. However, most of the research has been conducted on clinically relevant populations, and not on young, athletic men to enhance androgen response. Regardless, the mechanism responsible for much of the aromatase inhibition attributed to these products is attributed to the flavonoid content of these nutrients.

### 1.2. Flavonoids

Several compounds belonging to the class of flavonoids have been suggested to act as aromatase inhibitors [38]. Flavonoids are a group of natural substances with variable phenolic structures. They belong to a class of plant secondary metabolites and are widely found in fruits, vegetables, and certain beverages [42]. These natural products are well known for their beneficial effects on health due to their antioxidative, anti-inflammatory, anti-mutagenic, and anti-carcinogenic properties coupled with their capacity to modulate key cellular enzyme functions [42] including aromatase inhibition [43]. Most of the biological activities of flavonoids are attributed to a catechol group in their B-ring, 2,3-double bond conjugated with the 4-oxo function and a 3- (and 5-) hydroxy group that scavenge superoxide anions, hydroxyl, peroxyl, alkoxyl and the nitric oxide radicals, and by that eliminate lipid peroxidation [44].

There are numerous types of flavonoids found in various foods, providing potential anti-aromatase activity in many of the nutrients common in one’s diet. Apigenin is a flavonoid found in many fruits and vegetables, but commonly found in parsley, celery, celeriac, and chamomile tea [45]. Flavonoids are also found in beer. Xanthohumol-rich stout beer contains a high concentration of prenylflavonoids and is noted for its ability to inhibit aromatase activity [46]. Other flavonoids such as catechins exist in high concentrations in cocoa [47], prune juice [48], and Açaí oil [49]. Neves and colleagues [50] identified several flavonoids as equal or better aromatase inhibitors in comparison to the anti-aromatase drug aminoglutethimide, including flavones, flavanones, resveratrol, and oleuropein. Flavones and flavanones are found in many fruits and green tea, resveratrol is one of the main components of red wine, and oleuropein is found in olive oil. Several studies have reported high anti-aromatase activity in chrysin, a flavonoid present in high concentrations in honey and propolis [48,51,52]. However, others reported no change in testosterone concentrations in men consuming honey and propolis for 21 days [53]. Consuming foods or dietary supplements containing nutrients with aromatase inhibitors may provide an ergogenic effect by inhibiting the conversion of testosterone to estradiol, indirectly increasing testosterone concentrations. Unfortunately, most studies that have focused on the effect of various foods on aromatase inhibition have been primarily interested in the potential clinical use for treatment or prevention of various diseases such as breast cancer, and not on the ergogenic potential that they may have for competitive athletes. Additional research is needed to investigate the potential ergogenic effect aromatase inhibitors have on testosterone concentrations in athletic populations.

### 1.3. Other Nutrients

There have been other naturally occurring nutrients that have been suggested to have a potential anabolic effect by increasing testosterone concentrations. A multi-ingredient supplement consisting of indole-3-carbinol (an extract from cruciferous vegetables), chrysin, saw palmetto, and *Tribulus terrestris*, a plant of the *Caltrop family*, consumed in combination with the pro-hormones androstenedione and dehydroepiandrosterone increased FT concentrations to a greater extent in middle-aged men compared the pro-hormones only [54]. Others have examined the capability of the element boron on its testosterone-boosting capability [55,56,57]. Evidence does indicate significant elevations in testosterone concentrations in post-menopausal women [55] and in healthy men [56] following boron supplementation, but other examining weightlifters showed no difference between athletes supplementing with boron compared to placebo [57]. The mechanism suggested that enhancing testosterone concentrations from boron intake is related to boron’s role in the hydroxylation step during testosterone formation [58], and by its ability to decrease SHBG [59]. A decrease in SHBG levels would result in an increase in FT concentrations. Various foods such as fruits, tubers, wine, cider, beer, coffee, milk, dried and cooked beans, potatoes, and legumes contain the largest amounts of boron [60]. Additional research appears warranted regarding boron’s efficacy in increasing testosterone concentrations.

Phosphatidylserine (PS) has also been proposed to enhance the anabolic response to exercise. However, there is only limited human evidence supporting this hypothesis. PS is a phospholipid found in the cell membrane of a variety of tissues, including the brain, lungs, heart, liver, and skeletal muscle. The best dietary sources of PS are organ meats such as brain, liver, heart, and kidney. Fatty fish, meats, and white beans can also provide PS in smaller amounts. In a study on healthy, young men, Starks and colleagues [61] reported that PS supplementation significantly increased the testosterone to cortisol ratio during a bout of moderate intensity exercise on a cycle ergometer. Despite these positive results, there does not appear to be any additional research supporting the role of PS on changes in testosterone concentrations. However, other investigators examining another phospholipid (i.e., phosphatidic acid) reported an increase in both strength and muscle thickness in young, healthy men [62]. However, the mechanism that was suggested was related to the role that phosphatidic acid may have on stimulating the mTor protein signaling pathway and not to an augmented androgen response. However, the latter was not measured.

There appears to be an abundance of testosterone boosters that are marketed to the consumer. Balasubramanian and colleagues [63] recently examined the efficacy of the five top-ranked products and reported that the number of human studies conducted provided no definitive evidence for the efficacy of these products. Similarly, Clemesha and colleagues [64] reported that only ~25% of the 50 products they tested claiming to be testosterone boosters had scientific data to support their claims. This is an area of study that has much appeal to competitive strength/power athletes, but the evidence to support the use of these nutrients is often lacking.

## 2. Macronutrient Effects on Changes in Testosterone Concentrations

### 2.1. Low Energy Availability and Calorie Intake

Competitive athletes focusing on enhancing their athletic performance often strive to improve their body composition by increasing lean body mass and decreasing fat mass [65,66,67]. In the absence of appropriate guidance (e.g., consultation from a sport nutritionist), many athletes alter their dietary intake potentially creating an energy deficit, which is often associated with low energy availability [68]. Energy availability is defined as the difference between energy intake and energy expenditure, relative to an individual’s fat-free mass (FFM) [69]. Low energy availability may reduce the body’s energy reserves, limiting its ability to support normal physiological function needed to maintain optimal health [68]. For example, an athlete training at a high intensity or prolonged duration, while attempting to lose fat mass by reducing caloric intake, may cause a low energy availability. It is recommended that athletes have an energy availability of >45 kcal·kg FFM·day^−1^. Low energy availability is defined as <30 kcal·kg FFM·day^−1^ [69,70,71]. The impact of low energy availability on various physiological systems in the body is not the primary scope of this paper, instead, the focus is directed on the effect of low energy availability on circulating testosterone concentration and testosterone biosynthesis.

Several studies have demonstrated that a low energy availability can decrease LH concentrations, subsequently affecting testosterone synthesis. Initial investigations reported that a low energy availability (i.e., 13 kcal·kg FFM·day^−1^) significantly altered LH pulse frequency and amplitude [72]. Subsequent research, examining an even lower energy availability (i.e., 10 kcal·kg FFM·day^−1^) also reported significant decreases in LH production and LH pulse frequency [73]. Another investigation, comparing study participants experiencing a low energy availability of <30 kcal·kg FFM·day^−1^ to study participants with an energy availability of 45 kcal·kg FFM·day^−1^ reported a significant reduction in LH production and pulse frequency in the lower energy available group [74]. These studies clearly indicate the negative effect that low energy availability has on the hypothalamic-pituitary-gonadal axis.

Large energy deficits appear to negatively affect testosterone concentrations. Hu and colleagues [75] observed a significant decrease in testosterone concentrations when dietary macronutrient intake was reduced. Others have reported that a 40% reduction in total caloric intake was associated with significant decreases in circulating testosterone concentrations, despite a high percentage of the caloric intake being from protein sources [76]. Situations of low energy availability are reported in both endurance and strength/power athletes and are especially relevant in sports where competition is based upon weight class [77]. One investigation examining the effects of energy restriction and training volume on circulating testosterone concentrations reported a significant reduction in testosterone concentrations among physique athletes who increased their training volume, while being energy-restricted compared to physique athletes who maintained their regular diet and training volume [78] (described in Table 1). Additional research reported significant decreases in testosterone concentrations in long-distance runners who were categorized as “low energy available” (<30 kcal·kg FFM·day^−1^) compared to runners categorized as “moderate energy available” (30–45 kcal·kg FFM·day^−1^) [79]. Others have reported significant elevations in cortisol, decreases in testosterone, and a lower testosterone/cortisol ratio 24-h following an intense exercise session resulting in an energy deficit exceeding 400 kcal in male athletes [80]. These investigations have indicated that low energy availability has a deleterious effect on various physiological systems in the body, specifically the endocrine system.

**Table 1:** Effect of low energy availability and energy deficits on circulating testosterone concentrations.

Source	Participants	Duration	Intervention	Key Findings
[78]	*n* = 14 men Elite bodybuilders	11 weeks	Energy-restricted group (*n* = 7): decrease calories and increase energy expenditure through exercise Control group (*n* = 7): energy intake and training volume were maintained.	Significant decrease in TT in the energy-restricted group compared to the control group ES energy-restricted group = 0.49 ES control group = 0.07.
[76]	*n* = 34 Healthy adults (men and women)	31 days	40% energy deficit for all protein intake groups Group 1 (*n* = 11; 10 males, 1 female)—0.8 g∙kg^−1^∙day^−1^ Group 2 (*n* = 12; 10 males, 2 females)—1.6 g∙kg^−1^∙day^−1^ Group 3 (*n* = 10; 8 males, 2 females)—2.4 g∙kg^−1^∙day^−1^	Significant reduction in TT compared to the weight maintenance period in all groups ES group 1 = 3.38 ES group 2 = 0.39 ES group 3 = 0.36.
[79]	*n* = 24 males Elite distance runners	7 days	Low energy available group (*n* = 6): (<30 kcal·kg FFM·day^−1^) Moderate energy available group (*n* = 18): (30–45 kcal·kg FFM·day^−1^)	Significant reduction in TT in the low energy available group compared to the moderate energy available group ES = 1.3.

FFM = Fat free mass; TT = Total testosterone concentrations; ES = effect size. Effect size was estimated as (mean 2 − mean 1)/pooled standard deviation.

### 2.2. High-Fat Diets and Dietary Fats

For the hormonal system to function optimally, fat becomes an important macronutrient as it’s the backbone for steroid hormone production [81]. The minimum recommendation for dietary fat consumption should be not below 25% for both the general and athletic populations [82,83,84]. Cholesterol, a dietary fat component, is one of the building blocks for testosterone production [81]. Considering that high-fat diets (HFD) increase cholesterol levels, increases in dietary fat consumption have been suggested to potentially result in an increase in testosterone production [85]. Several studies have examined the effect of HFD on circulating testosterone concentrations [85,86,87]. One study examined the effect of ketogenic (KD) and non-ketogenic (NKD) diets on strength, body composition, and hormonal profile in resistance-trained men [84]. Both study groups consumed a diet high in fat (75% vs. 65% for the KD and NKD, respectively). The primary difference between the diets was that the KD group ingested a very low amount of dietary carbohydrates (5%) compared to the NKD (15%). Both groups participated in a 12-week resistance training program. Results indicated that both groups experienced a significant increase from baseline in both total testosterone (TT) and FT concentrations during all four assessment times (weeks 2, 4, 6, and 8) of the study. In addition, TT and FT concentrations at weeks 4, 6, 8 were significantly elevated compared to week 2. Although the investigative team did not employ a control group consuming a low-fat diet (LFD), the results do suggest that a HFD rich in cholesterol increased TT and FT in resistance-trained men regardless of the state of ketosis. A similar study [86] investigated the differences between KD and Western diets (WD) on body composition, strength, power, and hormonal profiles in resistance-trained men. In this study, a large difference in carbohydrate and fat consumption was noted between the two groups. The macronutrient profile for the WD group was 20%, 55%, and 25% for protein, carbohydrate, and fat, respectively, while for the KD group it was 20%, 5%, and 75%, respectively. Both groups participated in a 10-week resistance training program. Although both groups had similar increases in muscle thickness, lean body mass, and strength measures (1RM squat and bench press), only the KD group experienced a significant increase in TT concentrations. It has been suggested that increases in testosterone concentrations resulting from a KD are related to the high dietary intake of cholesterol [88]. Another study examining the effect of dietary nutrients on testosterone concentrations in resistance-trained individuals reported a significant correlation (r = 0.72) between resting testosterone concentrations and dietary fat consumption [83]. An investigation in women reported that a diet that included 40% of its calories from dietary fat was superior for increasing testosterone and estrogen concentrations compared to a diet whose total fat intake was 20% of its total caloric intake [89] (described in Table 2). A recent systematic review investigated the effect of a LFD on testosterone concentrations in men [85]. The LFD had a small-to-moderate effect on decreasing total testosterone concentrations compared to the HFD. Although evidence points to a positive effect of dietary fat on circulating testosterone concentrations, it should not be dismissed that fat is not the optimal fuel source for athletes participating in strength/power sports [83,90,91,92]. As such, sport nutritionists may hesitate in recommending high-fat diets.

### 2.3. Dietary Protein and Protein Supplements

Dietary protein intake has an important role in athletic performance. Skeletal muscle, the site for force production, is predominantly comprised of protein [93]. Dietary protein supports the replenishment of skeletal muscle protein as it is degraded or damaged by intense exercise [94,95]. Exercise and inadequate protein intake can result in a catabolic response of muscle. That is, if protein intake is not adjusted to meet the protein needs of the body, the ability to maintain or increase muscle mass and muscle performance becomes challenging. To maintain appropriate muscle protein balance, in which muscle protein synthesis equals or exceeds muscle protein breakdown, dietary protein intake for athletes is recommended to be between 1.6–2.2 g·kg^−1^·day^−1^ [96,97,98]. Although dietary protein has an important role in skeletal muscle size and strength adaptations, the influence of dietary protein intake on testosterone concentrations does not appear to have a major role, and the general recommendations of 1.6–2.2 g·kg^−1^·day^−1^ appear to be sufficient to optimize testosterone concentrations [94].

One type of protein that has received considerable attention regarding its effect on circulating testosterone concentrations is soy protein. Although soy protein consumption has been demonstrated to have significant benefits on strength performance following 12 weeks of resistance training [99], its role in androgen biology has made it a topic of interest. This interest is related to the understanding that soybeans contain biologically relevant amounts of isoflavones [100]. Isoflavones are also referred to as phytoestrogens and are compounds that bind to estrogen receptors, causing a cascade of events that exert estrogen-like effects [101,102]. The popularity of soy protein use among vegan athletes has resulted in a focus on its effect on sport performance and changes in testosterone concentrations. Studies on male rodents have resulted in conflicting results. Some investigations have indicated that rodents fed soy protein exhibited lower serum testosterone concentrations [103,104], while others reported no negative effects on circulating testosterone [105,106]. A meta-analysis examining the effect of isoflavones and soy protein intake on testosterone concentrations and SHBG in men concluded that there was no negative effect of soy protein intake on TT, FT, or SHBG [107]. However, subsequent research continues to still raise the issue that soy protein consumption can result in reductions in resting testosterone concentrations and response to exercise. Kraemer and colleagues [108] examined the effect of soy protein supplementation on the testosterone and estradiol response in healthy, resistance-trained men. Using a cross-over design, participants were randomized into three groups: whey protein supplement (WPS), soy protein supplement (SPS), and maltodextrin (placebo, PLA). Participants were provided each treatment for 14 days. Following the supplementation period, participants performed an acute resistance exercise session consisting of 6 sets of 10 repetitions in the squat exercise at 80% of the participant’s one repetition maximum. Testosterone and estradiol concentrations were examined at various time points during and following the acute exercise session. Although testosterone concentrations were significantly elevated from baseline during and up to 5-min post-exercise, only the WPS and PLA trials resulted in significant increases in testosterone concentrations at the 15- and 30-min post-exercise measures. Additionally, testosterone concentrations were significantly lower during SPS compared to both WPS and PLA during the post-exercise period. No significant differences were noted in estradiol concentration between the three groups at any of the time points. Subsequent research, using the same three groups, albeit for 12 weeks and combined with resistance exercise, also reported no significant pre- or post-training changes in serum estradiol concentrations in any of the study groups [109]. Although no significant differences were noted in TT concentrations in both the SPS and PLA groups, an increase in serum TT was observed in the WPS group. In addition, muscle androgen-responsive mRNA was increased in all groups as a result of the training stimulus (significant main effects for time), but no significant differences were observed between the three groups. The results from these studies do not provide any conclusive evidence regarding soy protein having a negative effect on testosterone production. However, they are consistent in demonstrating that whey protein supplementation may be the superior protein supplement for enhancing the testosterone response to training. It is also important to note that protein supplementation regardless of its source (soy or whey), when combined with resistance training can result in a significant increase in lean body mass [110].

**Table 2:** Effect of fat and protein intake on changes in circulating testosterone concentrations.

Source	Participants	Duration	Intervention	Key Findings
[89]	*n* = 48 Premenopausal healthy women	14 weeks	HFD group (*n* = 24): 40% fat LFD group (*n* = 24): 20% fat	Significant decrease in TT concentrations Significant decrease in estrone concentrations Significant decrease in SHBG concentrations
[108]	*n* = 10 Healthy resistance-trained men (crossover design)	10 weeks	WPI (*n* = 10) SPI (*n* = 10) Placebo (*n* = 10)	Significant increase in the TT response to resistance exercise test for all groups. Significantly lower TT concentrations in SPI group post-resistance exercise compared to both WPI and placebo groups.
[109]	*n* = 47 Healthy college-aged men performing a resistance training program	12 weeks	WPI (*n* = 17) SPI (*n* = 15) Placebo (*n* = 15)	No significant differences in TT concentrations between SPI and placebo groups ES SPI = 0.04 ES PLA =0.15. Significant increase in serum TT concentrations compared to baseline in the WPI group (ES = 0.63). Significant pre to post effects in all groups for muscle androgen-responsive mRNA, but no significant differences noted between groups. ES PLA = 0.05 ES SPI = 0.28 ES WPI = 0.59.
[86]	*n* = 25 Resistance-trained men	11 weeks	Ketogenic diet (*n* = 13) Western diet (*n* = 12)	Significant increase in TT concentrations in the ketogenic diet compared to western diet group ES = 0.65.
[111]	*n* = 18 Experienced, resistance-trained, middle-aged men	8 weeks	Ketogenic diet (*n* = 9) Non-ketogenic diet (*n* = 9)	Significant increase in both TT and FT in both the ketogenic and non-ketogenic groups. ES, KD for TT = 1.98, TF = 1.66 ES, NKD for TT = 2.27, TF = 2.31

FT = free testosterone; HFD = high-fat diet, LFD = low-fat diet; SHBG = sex hormone binding globulin; SPI = Soy protein isolate; TT = total testosterone; WPI = whey protein isolate; ES = effect size. Effect size was estimated as (mean 2 − mean 1)/pooled standard deviation.

## 3. Micronutrient Effects on Testosterone Concentrations

### 3.1. Vitamin D

Vitamin D is a micronutrient that also acts as a prohormone [112]. Vitamin D has garnered considerable attention in the general and competitive athletic populations, primarily due to its role on various physiological systems in the body, and the effect that vitamin D deficiency has on many diseases [113,114]. Vitamin D has two biological forms, vitamin D3 (cholecalciferol), and vitamin D2 (ergocalciferol). Vitamin D3 is the most bioavailable and most supplemented form of Vitamin D and is synthesized in the skin upon exposure to sunlight. The specific physiological effects of vitamin D and its specific mechanisms are beyond the scope of this paper, but readers are encouraged to explore this elsewhere [115,116]. In brief, vitamin D, whether it is synthesized endogenously or consumed as a food or supplement, undergoes hydroxylation to become active. The first hydroxylation step occurs in the liver where vitamin D is converted to 25-hydroxyvitamin D [25(OH)D]. The second hydroxylation step is performed primarily in the kidney to form 1,25-dihydroxyvitamin D3, also referred to as 1,25-dihydroxycholecalciferol, which is the biologically active form of vitamin D [117,118]. The United States Institute of Medicine has indicated that the range for vitamin D concentrations should be between 25–50 nmol·L^−1^ [119]. Studies on athletes have suggested that the cut-off for vitamin D deficiency should be > 30 nmol·L^−1^ or even higher [112,120,121].

The vitamin D receptor, which plays a central role in the biological action of the vitamin, has been observed in reproductive tissues such as the ovaries, prostate, and testes, as well as in human sperm [122,123,124,125]. Vitamin D receptors are present on the Leydig cells within the testes, where the synthesis of testosterone from cholesterol occurs [7], suggesting an important role of vitamin D on testosterone synthesis. Men with vitamin D deficiency have exhibited significantly lower testosterone concentrations compared to men with normal vitamin D concentrations [126]. Significant associations were also noted between vitamin D concentrations and circulating testosterone and SHBG concentrations, as well as the free androgen index [126]. These findings are consistent with subsequent investigations reporting significant correlations between vitamin D and testosterone concentrations [126,127,128].

Athletes in general are at a higher risk for vitamin D deficiency, especially athletes participating in indoor sports [121,129,130,131]. Vitamin D supplementation is a potential option to maintain normal vitamin D status, but also to potentially increase testosterone concentrations. A double-blind, randomized placebo-control trial of 54 males reported that the group receiving a daily supplementation of 83 μg (3332 IU) of vitamin D for 12 months experienced significant increases in circulating 25-hydroxyvitamin D, TT, and FT concentrations compared to the placebo group [132] (described in Table 3). Although it has been suggested that the daily dose of vitamin D supplementation for athletes should be 5000 IU·day^−1^ for improving performance and restoring vitamin D levels [120,133,134], no consensus exists regarding the optimal range for vitamin D levels [119]. Furthermore, the effect of vitamin D supplementation on altering resting testosterone concentrations is still not well understood and requires further research.

### 3.2. Zinc

Zinc is a mineral that influences and interacts with many biological systems, especially the endocrine system [16]. Zinc has an important role in immune system function and in modulating inflammatory processes [135,136,137,138]. While zinc can be found in many food sources, the more bioavailable form of zinc can be found in animal tissues [139,140,141,142]. The daily recommended intake for zinc is between 14–40 mg·day^−1^ [143]. The physiological role of zinc regarding testosterone biology is related to its requirement in the synthesis and secretion of LH. As previously discussed, LH stimulates testosterone synthesis in the Leydig cells [6,14,144]. Zinc is also important in the conversion of testosterone to DHT [144]. DHT is converted from testosterone by the enzyme 5α—reductase in the cytoplasm of the cell. DHT is primarily found in peripheral tissues such as prostate, skin, hair follicles, and the liver [6,145]. As discussed earlier, DHT is thought to have a stronger androgenic affect than testosterone due to its four-times greater binding affinity for the androgen receptor than testosterone and a three-times slower dissociation rate than testosterone [146,147,148]. DHT has a vital role in the sexual development of males and sexual differentiation of organs and promotes prostate growth; male pattern baldness; and body, facial, and pubic hair growth [6,145,149].

Zinc also has an indirect role in testosterone synthesis. Zinc is required for normal function of angiotensin-converting enzyme (ACE), a zinc-dependent dicarboxypeptidase, which has a zinc binding site in its cyclitic domain [150,151]. ACE is reported to increase LH production in pituitary, thus impacting androgen production [152]. Zinc deficiency can impair testosterone synthesis and has been demonstrated to correlate with reductions in testosterone concentrations [153,154,155]. Competitive athletes appear to be at a greater risk for zinc deficiency compared to the general population [156,157]. Considering that zinc deficiency appears to be related to hypogonadism, efforts to maintain zinc levels within normal ranges appears important. Several studies have shown that zinc supplementation can restore testosterone concentrations to their normal physiological range [153,154,158]. One study examined the effect of zinc supplementation on both TT and FT concentrations in healthy young adults before and after an exhaustive exercise protocol [159]. Study participants were supplemented with zinc sulfate (3mg·kg·day^−1^) for four weeks. Investigators reported that zinc supplementation increased both TT and FT concentrations prior to and following the exhaustive exercise protocol compared to pre-supplementation results. In contrast, others reported no difference in either the TT or FT response to exhaustive exercise between male cyclists supplemented with zinc sulfate (30 mg) for four weeks compared to a placebo-controlled group [160]. Although zinc has an important role in the regulation of testosterone production, long-term studies in competitive athletes have not been conducted. Whether zinc supplementation is effective only during periods of zinc deficiency or whether it can augment normal testosterone concentrations regardless of baseline concentrations is not well understood.

### 3.3. Magnesium

Magnesium is one of the most abundant minerals in the body. It has an important role in various biological systems including protein synthesis, cellular energy production, cell growth, and reproduction [161]. From an athletic performance perspective, magnesium is involved in skeletal muscle function and energy production, suggesting a possible ergogenic effect [162]. The recommended dietary allowance for magnesium intake for men is between 400 to 420 mg·day^−1^ and 310 to 320 mg·day^−1^ for women [163]. Several studies have reported that athletes do not consume enough magnesium from their diet, resulting in a greater risk for magnesium deficiency [164,165,166,167]. Several investigations have reported a relationship between magnesium and testosterone concentrations [168,169,170]. One study indicated that magnesium supplementation in young healthy men in combination with a four-week endurance training program increased both FT and TT concentrations at rest and following exhaustive exercise [171]. An additional study conducted on nearly 400 older adult men reported a significant correlation between magnesium status and testosterone concentrations (r = 0.20, *p* < 0.05) [169]. The mechanism responsible for this relationship has yet to be elucidated. However, it is possible that it may be more indirect than direct. Magnesium is known to have a role in decreasing oxidative stress and inflammation [172,173,174]. Considering that testosterone concentrations can be strongly influenced by oxidative stress [175], it is possible that magnesium’s role in decreasing oxidative stress may provide the stimulus to maintain testosterone concentrations during periods of oxidative stress. A strong positive correlation has been reported between total antioxidative capacity and testosterone concentrations (r = 0.807) [175]. Magnesium has an important role in maintaining antioxidant capacity and controlling oxidative stress [172,173,174]. Magnesium deficiency has been demonstrated to increase production of oxygen free radicals, increase oxidative tissue damage, decrease antioxidant enzyme activity, decrease cellular antioxidant levels, and increase oxygen peroxide production [176,177,178]. In contrast, normal magnesium levels can prevent oxygen radical formation by removing free radicals and inhibiting xanthine oxidase and nicotinamide adenine dinucleotide phosphate (NADPH) oxidase elevations [179].

Magnesium deficiency has also been associated with low-grade systemic inflammation [172,180], and has been shown to increase pro-inflammatory cytokines: tumor necrosis factor-alpha (TNF-α) and interleukin 1 (IL-1) [180,181,182]. Low-grade chronic inflammation has been shown to decrease testosterone concentrations by suppressing testosterone secretion from Leydig cells, resulting in both an inhibitory effect on LH secretion and reduced LH sensitivity at the Leydig cell [183,184]. Increases in TNF-α activates nuclear factor κB (NF-κB), a transcription factor that governs the expression of early-response genes involved in cellular responses to a wide range of signals [185]. NF-κB inhibits the activation of steroidogenic-enzyme genes such as Nur77 and SF-1, which regulate steroidogenesis (biosynthesis of testosterone from cholesterol) in the Leydig cells [184]. Rochelson and colleagues [186] demonstrated, through an in vitro examination, that magnesium sulfate can reduce the nuclear translocation of NF-κB. Others have demonstrated that magnesium supplementation can reduce inflammatory status and decrease levels of TNF-α and IL-1 [180,187].

Magnesium also appears to reduce the binding of testosterone to SHBG [188]. Most circulating testosterone is bound to SHBG; however, the bioavailability of testosterone is related to the free testosterone concentrations, which is only a fraction of circulating testosterone [189]. Magnesium appears to bind to SHBG resulting in the blocking of testosterone’s ability to bind to SHBG, subsequently enhancing testosterone bioavailability. Magnesium deficiency appears to increase testosterone binding to SHBG, potentially decreasing its bioavailability [188]. Whether magnesium supplementation is effective in augmenting testosterone synthesis as an anabolic agent is not well understood.

**Table 3:** Effect of micronutrient intake on circulating testosterone concentrations.

Source	Participants	Duration	Intervention	Key Findings
[159]	*n* = 10 Healthy college-aged men	4 weeks	Zinc supplementation group (zinc sulfate 3 mg·kg·day^−1^)	Significantly elevation of TT and FT before and after an exhaustive exercise protocol compared to pre-supplementation. ES before the exhaustive exercise protocol TT = 0.59 FT = 1.32 ES after the exhaustive exercise protocol TT = 0.82, FT = 3.32
[132]	*n* = 54 Healthy overweight men	12 months	Vitamin D supplementation group (*n* = 31): 83 μg·day^−1^ (3332 IU) Placebo group (*n* = 23)	Significant increase in TT and FT in the vitamin D group compared to baseline. ES for TT in the VD group = 0.63 ES for FT in the VD group = 0.54
[160]	*n* = 32 Male cyclists	4 weeks	Zinc supplementation group n = 8 (30 mg·day^−1^) Selenium supplementation n = 8 (Se, 200 μg sodium selenite) zinc–selenium *n* = 8 (Zn–Se, 30 mg zinc sulfate–200 μg selenium selenite) Placebo group *n* = 8 (30 mg·day^−1^)	No significant differences between groups for TT and FT before and after the exhaustive exercise test.
[171]	*n* = 30 Healthy college-aged men participating in an aerobic -training program	4 weeks	Magnesium supplementation + training group *n* = 10 (MgSO_4_ 10 mg·kg·day^−1^) Magnesium supplementation group only *n* = 10 Training group only *n* = 10	Significant increase in total and free testosterone concentrations before and after exhaustive exercise in the group who supplemented with magnesium and trained compared the other groups. ES before TT = 0.37 ES after TT = 0.46 ES before FT = 1.11 ES after FT = 0.45

FT = Free testosterone; TT = total testosterone; ES = effect size. Effect size was estimated as (mean 2 − mean 1)/pooled standard deviation.

## 4. Summary

In summary, this article discussed several nutrients that have been proposed to have anti-aromatase activity. Although evidence has been presented supporting the benefits of certain nutrients, the evidence supporting most of the nutrients suggested to influence anti-aromatase activity remain largely inconclusive. Much of this issue is related to the small sample sizes found in many of these papers, and the limitations that are associated with examining trained and athletic populations. More effort appears to have been focused on the effects of energy intake and manipulating macronutrient composition, specifically protein and fat composition on changes in circulating levels of testosterone at rest and in response to various exercise stresses. Evidence is consistent in demonstrating that low energy intake negatively impacts testosterone concentrations that may affect human performance. In addition, certain vitamins and minerals have important roles in testosterone synthesis. The importance of supplementing these vitamins and minerals appears to become efficacious when the body becomes deficient in these specific micronutrients. However, evidence supporting any benefits of supplementing with these micronutrients to augment testosterone concentrations is lacking.
